# Phonon controlled transmission properties of metasurfaces under strong light–matter coupling

**DOI:** 10.1515/nanoph-2025-0123

**Published:** 2025-05-26

**Authors:** Zengshun Jiang, Kewei Sun, Yang Zhao, Konstantin Dorfman

**Affiliations:** State Key Laboratory of Precision Spectroscopy, 12655 East China Normal University, Shanghai 200241, China; School of Science, Hangzhou Dianzi University, Hangzhou 310018, China; School of Materials Science and Engineering, Nanyang Technological University, Singapore 639798, Singapore; Center for Theoretical Physics and School of Physics and Optoelectronic Engineering, Hainan University, Haikou 570228, China; Himalayan Institute for Advanced Study, Unit of Gopinath Seva Foundation, MIG 38, Avas Vikas, Rishikesh 249201, Uttarakhand, India

**Keywords:** metasurface, strong light-matter coupling, phonons, time-dependent variation, Davydov’s *Ansätze*

## Abstract

In the strong light–matter coupling regime, the transmission properties of metasurfaces have a remarkable similarity to those of typical two-level systems. In this work, we explore the absorption spectra of a metasurface coupled to a quantum phonon bath using the time-dependent variational principle and the flexible multi-D2 Davydov trial states. In the weak light–matter coupling regime, phonon coupling has minimal impact on system dissipation. However, in the strong coupling regime, it significantly influences dissipation dynamics. Additionally, a phonon bath with a selected number of strongly coupled modes near the phonon center line substantially narrows the absorption spectrum linewidth by controlling dissipation through a few phonon channels. These findings demonstrate the critical role of the phonon bath in shaping metasurface transmission properties, offering a promising approach for the precise engineering of metasurfaces.

## 1 Introduction

Cavity quantum electrodynamics (QED) is a well-established field of research that explores the fundamental interactions between light and matter within an optical cavity, where the quantum properties of photons play a central role. In this context, the cavity serves as a resonator, confining light to a small volume and enhancing the interaction between photons and quantum emitters, such as atoms, molecules, or quantum dots. In diverse scenarios, such as singlet fission [1], [2], chemical reactions, and electron transfer processes in cavities [3], significant attention has been given to polariton formation and the strong coupling between cavity modes and molecular/atomic excitons [4], [5], [6], [7], [8].

Strong light–matter interactions can be achieved by coupling the photonic modes of microcavities to various electronic/vibrational degrees of freedom, resulting in mixed electronic-vibrational-photonic states. It is essential to be in the strong coupling regime in order to observe coherent phenomena such as Rabi oscillations [9], coherent energy transfer, quantum information processing [10], [11], [12], [13], and single-atom lasers [14]. The underlying principle of strong light–matter coupling relies on the relationship between coupling strength and system dissipation, with strong coupling occurring only when the coupling strength exceeds the dissipation rate. To achieve strong light–matter coupling, two common approaches are typically employed. The first involves leveraging the significant reduction in system dissipation at low temperatures, ensuring that dissipation remains much weaker than the coupling. This approach is characteristic of traditional atomic [15] and solid-state microcavity [16], [17] systems operating at low temperatures and under ultra-high vacuum conditions. The second approach focuses on enhancing the light–matter coupling strength to overcome significant dissipation at room temperature, such as the use of plasmonic cavities and quantum emitters under strong coupling [18], [19], [20], [21]. Despite substantial losses in plasmonic structures, the high concentration of organic molecules and large oscillation intensities facilitate achieving the strong coupling regime. Furthermore, systems containing metal nanoparticles may exhibit resonant behavior of electromagnetic fields, which can be tuned in frequency and space by carefully selecting the geometry of the nanoparticles and their respective positions. Regular arrays of metal nanoparticles can exhibit extremely narrow light extinction profiles, known as surface lattice resonances (SLRs), due to collective resonances [22], [23], [24], [25], [26], thus greatly reducing the dissipation of the system. In recent years, numerous exciting advancements have demonstrated strong coupling between plasmonic or dielectric cavities and light at room temperature using SLR metasurfaces [27], [28], [29], [30].

In conventional metasurface studies, the focus has been primarily on the electronic states of metasurface materials and the photonic modes of the light field, with limited attention given to environmental effects, such as the phonon bath. The bath effects were mostly discussed in the context of nonlinear Raman processes [31], [32] or Brillouin scattering [33]. Furthermore, the environment has profound effects on the transmission properties of metamaterials. Conventionally, metasurfaces have a low Q factor due to strong free-space coupling combined with high radiation losses in the visible and near-infrared wavelength range, which results in a broad absorption linewidth. Therefore, engineering the environment by manipulating the coupling and the spectral density of the phonon bath which embeds the metasurface can provide an avenue for controlling the transmission and absorption spectral properties of metasurfaces.

In this work, we first introduce a QED model in which a simple Jaynes–Cummings (JC) Hamiltonian is coupled to a phonon reservoir. We compute numerically accurate many-body dynamics of the system by utilizing the time-dependent multiple Davydov Ansatze method, a numerically accurate many-body quantum dynamics method developed by Zhao and coworkers [34]. Simultaneously, the absorption spectrum of SLR metasurface is simulated by using the finite-difference time-domain (FDTD) method, representing a practical experimental system. This model is employed to investigate the effects of phonon modes and phonon coupling strengths on the linewidth of the absorption spectrum. It is found that the coupling strength of phonons, the proportion of strong coupling phonon modes relative to the total phonon modes, and the phonon center frequency significantly affect the dissipation dynamics of the system, resulting in significant changes in the absorption spectrum linewidth.

## 2 Models and methodology

**Figure 1:**
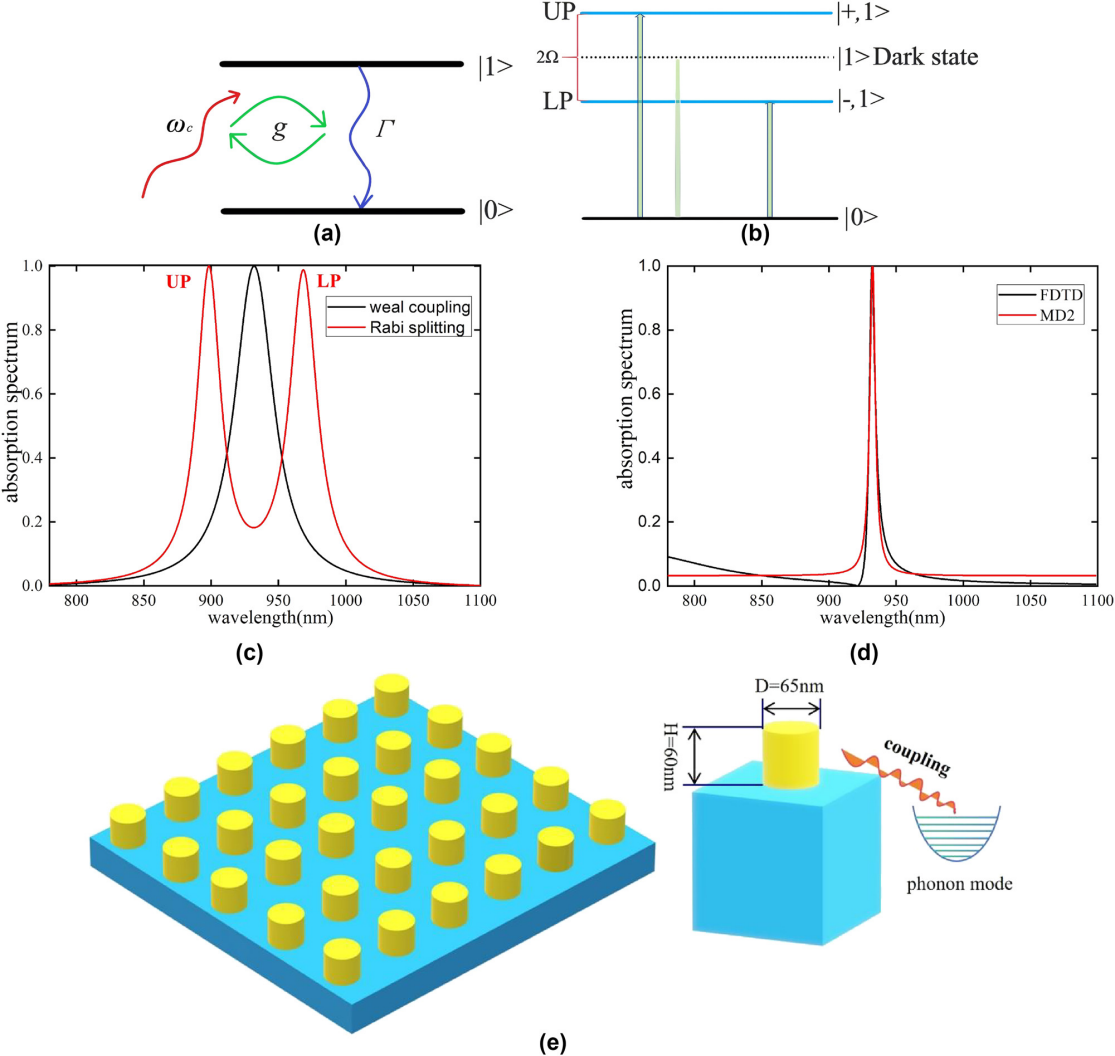
Light–matter interactions. (a) A schematic diagram of a two-level system interacting with a light field. (b) Strong coupling leads to the generation of dressed states. (c) Rabi splitting due to strong coupling. (d) Simulation results by FDTD and MD2A in the weak coulping regime. (e) Schematic diagram of the simulated metasurface. And an antenna coupling with phonon mode.

A metasurface system interacting with linearly polarized light can be represented by a JC model, expressed as follows:

(1) $${\hat{H}}_{\text{JC}}=\hbar \omega_{0}{\hat{\sigma }}^{†}{\hat{\sigma }}^{-}+\hbar \omega_{C}{\hat{a}}^{†}\hat{a}+\hbar g\left({\hat{a}}^{†}{\hat{\sigma }}^{-}+\hat{a}{\hat{\sigma }}^{†}\right)$$

where *g* is the strength of coupling between the metasurface open cavity mode and the two-level system, *ℏω*_*C*_ is the photon energy, *ω*_0_ is the transition frequency from the ground state to the excited state of the two-level system, ${\hat{a}}^{†}\left(\hat{a}\right)$ denotes the cavity-mode creation (annihilation) operators, and $\sigma^{†}=\left|0\right\rangle \left\langle 1\right|$ ( $\sigma^{-}=\left|1\right\rangle \left\langle 0\right|$ ) is the raising (lowering) Pauli operator of the system. The coupling strength can be expressed as $g=\Omega \propto \sqrt{\frac{\hbar \omega_{c}}{\varepsilon_{r}V}}\sqrt{f}$ , where *ɛ*_*r*_ is the relative dielectric constant, *V* is the mode volume, Ω is the Rabi frequency, and *f* is the dipole strength (*f* ∼ *μ* ^2^, *μ* is the transition dipole moment of the two-level system). A comparison of *g* with the decay rate Γ of a coupled two-level system will determine whether the system is in the weak coupling regime (*g* < Γ) or the strong coupling regime (*g* > Γ) (Figure 1(a)).

For *g* < Γ, the system dynamics exhibit a clear dissipation effect, with the decay rate of the system and the linewidth of the absorption spectrum being determined by Γ [35], [36], which is expressed as

(2) $$\Gamma =2\Omega^{2}D$$

Here $D=\frac{1}{\pi }\frac{\omega_{C}/2Q}{{\left(\omega -\omega_{C}\right)}^{2}+{\left(\omega_{C}/2Q\right)}^{2}}$ is the density of states at the transition frequency *ω*, which is the Purcell factor [37] within the framework of QED, and *Q* is cavity quality factor. If the coupling becomes stronger (the system is still in a weak coupling regime), the linewidth will increase with the increase of Ω. And it can be clearly observed that the values of *g* and Γ can be determined through Ω.

In the strong light–matter coupling regime (*g* > Γ), the energy exchange between the cavity and the two-level system is much faster than the dissipation process, which leads to the emergence of two peaks in the dressed states, also called exciton polariton states (Figure 1(b)), whose energies are given by

(3) $$E_{\pm }=1/2\left(\omega_{C}+\omega_{0}\right)\pm 1/2\sqrt{{\left(\omega_{C}-\omega_{0}\right)}^{2}+4g}$$

where *E*_±_ denotes the energies of the two bright states: the upper polariton (UP) and the lower polariton (LP), as showed in Figure 1(b). The energy separation between the two eigenstates corresponds to Ω. With the resonant conditions *ω*_*C*_ = *ω*_0_ ≡ *ω*, one has *E*_±_ = *ω* ± *g*, so Ω = 2*g*. The absorption peak of the system will be split into two peaks, corresponding to the Rabi splitting (Figure 1(c)). While the corresponding decay rate [35] becomes

(4) $$\Gamma_{s}=\frac{3}{2}\Gamma$$

The substrate of a metasurface is typically composed of crystalline materials, where the phonon environment affects the absorption spectrum. The total Hamiltonian [10], [38], [39] which includes the phonon bath, can be expressed as

(5) $${\hat{H}}_{\text{tot}}={\hat{H}}_{\text{JC}}+\underset{k}{\sum }\hbar \omega_{k}{\hat{b}}_{k}^{†}{\hat{b}}_{k}+\underset{k}{\sum }\hbar \omega_{k}\lambda {\hat{\sigma }}^{†}{\hat{\sigma }}^{-}\left({\hat{b}}_{k}^{†}+{\hat{b}}_{k}\right)$$

where *ω*_*k*_ and ${\hat{b}}_{k}^{†}\left({\hat{b}}_{k}\right)$ are the frequency and creation (annihilation) operator of the *k*th phonon mode, respectively, and *λ* is the diagonal system-phonon coupling strength, which is affected by a variety of factors such as material, temperature [40], stress [41], and nanostructure [42], [43]. In general cases, the dispersion relation of optical phonons is nonlinear and cannot be approximated linearly. In exceptional scenarios, such as in low-dimensional materials (a V-shape LO branch is founded in h-BN/Cu foil extracted from EDC [44]), or in specific theoretical frameworks [45], the linear assumption may be used as a simplification tool. The approximation was adopted to simplify the calculations and more effectively reveal the essential role of phonons in strong light–matter interactions. Therefore, in this work, we adopt a linear optical phonon dispersion, namely,

(6) $$\omega_{k}=\omega_{k0}\left[1+\kappa \left(\frac{2\left|k\right|}{\pi }-1\right)\right]$$

where *ω*_*k*0_ denotes the central energy of the phonon band, 0 ≤ *κ* ≤ 1 is the dimensionless bandwidth, *k* = 2*π l*/*N*(*l* = −*N*/2 + 1, …, *N*/2) is the phonon wavevector. Since the excitation number operator

(7) $${\hat{N}}_{\text{ex}}={\hat{a}}^{†}\hat{a}+{\hat{\sigma }}^{†}{\hat{\sigma }}^{-}$$

commutes with the Hamiltonian ${\hat{H}}_{\text{tot}}$ and the number of excitations is thereby conserved, we restrict our consideration to singly excited states $\left\langle {\hat{N}}_{\text{ex}}\right\rangle =1$ . In order to deal with the complex dressed electron, the cavity mode and phonon degrees of freedom, we will employ the time-varying hierarchy of Davydov’s *Ansätze* method to accurately solve the multidimensional Schrödinger equation with the total Hamiltonian. The multi-D2 *Ansätze* (MD2A) with multiplicity *M* are given by

(8) $$\left|D_{2}^{M}\left(t\right)\right\rangle =\underset{n}{\sum }\left|n\right\rangle \overset{M}{\underset{m=1}{\sum }}A_{mn}\left(t\right)e^{\left(\underset{k}{\sum }f_{mk}\left(t\right)b_{k}^{+}-H.c.\right)}|0\rangle_{\text{ph}}$$

where $\left|n\right\rangle$ is the nth global electronic state of the two-level system, ${\left|0\right\rangle }_{\text{ph}}$ is the phonon vacuum state, H.c. represents the Hermitian conjugate, *A*_*mn*_(*t*) are the amplitudes of the excited states, and *f*_*mk*_(*t*) are the harmonic mode displacements for the *m*th coherent state and the *k*th mode.

In terms of the multi-D2 parameters, the time evolution of the photon-mode population is evaluated as follows:

(9) $$P_{\text{ph}}\left(t\right)=\overset{M}{\underset{m,m^{\prime }=1}{\sum }}A_{m1c}^{*}\left(t\right)A_{m^{\prime }1c}^{*}\left(t\right)S_{mm^{\prime }}\left(t\right)$$

where

$$\begin{array}{cc} S_{mm^{\prime }}\left(t\right) & =\exp{\underset{k}{\sum }\left\{-(\left|f_{mk}\left(t\right)\right|\right.}^{2}+{\left|f_{m^{\prime }k}\left(t\right)\right|}^{2})/2+\left.f_{mk}^{*}\left(t\right)f_{m^{\prime }k}\left(t\right)\right\} \end{array}$$

is the Debye–Waller factor. And the autocorrelation function *F*(*t*) based on the multi-D2 *Ansätze* is defined by

(10) $$F\left(t\right)=\left\langle {\hat{a}}^{†}\left(t\right)\hat{a}\left(0\right)\right\rangle$$

The corresponding multi-D2 expression for the cavity absorption spectrum reads

(11) $$\begin{array}{cc} F\left(\omega \right) & =\frac{1}{\pi }R\int_{0}^{\infty }F\left(t\right)e^{-\left(\gamma^{\prime }-i\omega \right)t}dt\\ & =\frac{1}{\pi }R\int_{0}^{\infty }dt\sum_{m,m^{\prime }=1}^{M}A_{m^{\prime }1c}^{*}\left(0\right)A_{\mathrm{m1c}}\left(t\right)e^{-\left(\gamma^{\prime }-i\omega \right)t}\\ & \;\times e^{\sum_{k}-\frac{1}{2}({\left|f_{mk}(t)\right|}^{2}+{\left|f_{m^{\prime }k}(0)\right|}^{2})+{f^{*}}_{m^{\prime }k}(0)f_{mk}(t)} \end{array}$$

where *γ*′ is the electronic dissipation factor (corresponds to Γ under different conditions). Although the formula for calculating absorption spectra differs from that in Reitz’s work [46], the methodologies are fundamentally equivalent: whereas we employ photon population dynamics, Reitz utilizes excited-state population evolution. Figure 1(d) shows the simulation results from FDTD and MD2A (see Supplementary Material for details). Using Eq. (2) and the definition of *g*, we can determine the parameter *g* that needs to be fitted, which facilitates our subsequent simulations. A schematic diagram of the metasurface and antenna is also shown in Figure 1(e).

## 3 Results and discussions

The system dynamics are calculated using the numerically accurate multi-D2 method. A multiplicity of *M* = 20 in the multi-D2 method ensures result convergence, as validated through comparison with previous studies. All calculations incorporate 11 phonon modes, and a dimensionless phonon bandwidth *κ* = 0.5 is used. Usually, a realistic bath spectral density determines the number of strongly coupled phonon modes that strongly couple with the system. Similar to the work of Sun et al. [10], the remaining modes exhibit weak coupling and can be neglected in our model. To simplify the calculations, only 11 phonon modes was selected for the simulation here.

### 3.1 Weak coupling with the cavity mode

**Figure 2:**
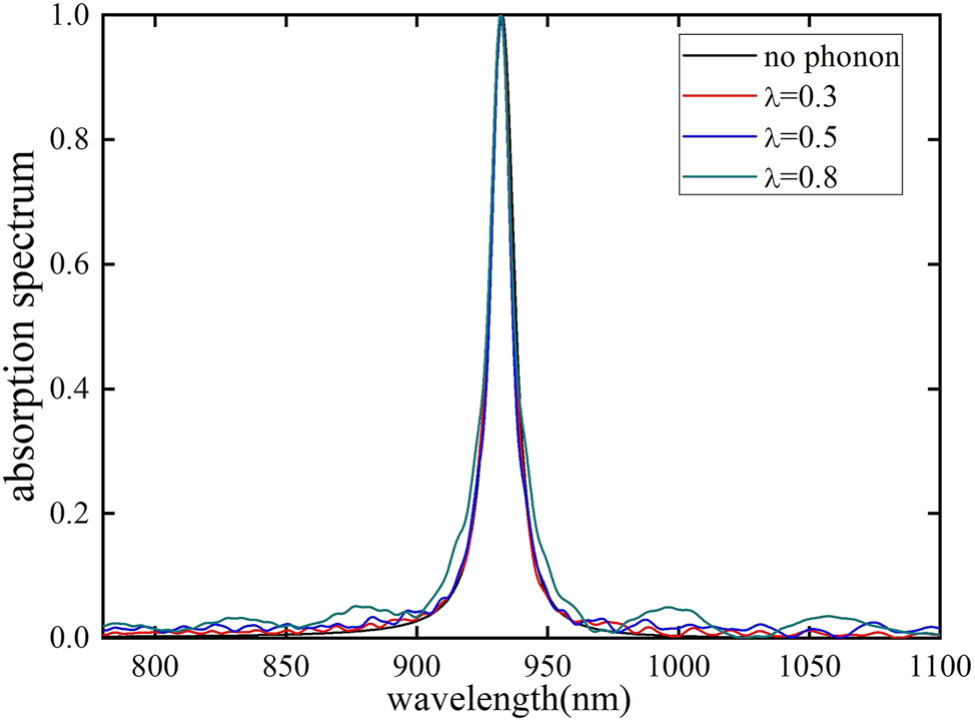
The absorption spectrum for difference phonon coupling strength under weak coupling with cavity. Phonon coupling has a minimal impact on the linewidth of the absorption spectrum.

In the case of weak light–matter coupling, a coupling coefficient of *g* = 0.005 eV and a center frequency of phonons *ω*_*k*0_ = 0.0496 eV are used. It is found that since the energy range of phonons is much smaller than the decay rate, its effect is not pronounced in the transmission of the weak light–matter coupling system, such as the Patcharanam–Berry phase (PB) metasurface. However, in the SLR metasurface system, the linewidth is significantly reduced due to the collective resonances, enabling the phonons’ influence to show up in the spectrum. Due to the low energy exchange efficiency between light and matter under weak light–matter coupling conditions, atoms cannot be effectively excited. However, once excited, the anharmonic coupling between the excited state and phonons leads to significant linewidth broadening [47]. This also explains why, in the regime of weak light–matter interactions, phonons have a relatively minor impact on the linewidth. As the phonon coupling strength increases, the linewidth is broadened slightly which is consistent with Reitz’s work [46]. This partially explains why the linewidth obtained from simulations is often narrower compared to experimental results in many systems. The absorption spectrum linewidth exhibits a modest increase with stronger phonon coupling, suggesting that phonon coupling has a minimal effect in the weak cavity-system coupling regime (see Figure 2).

### 3.2 Strong coupling with the cavity mode

#### 3.2.1 Effect of phonon coupling strength

**Figure 3:**
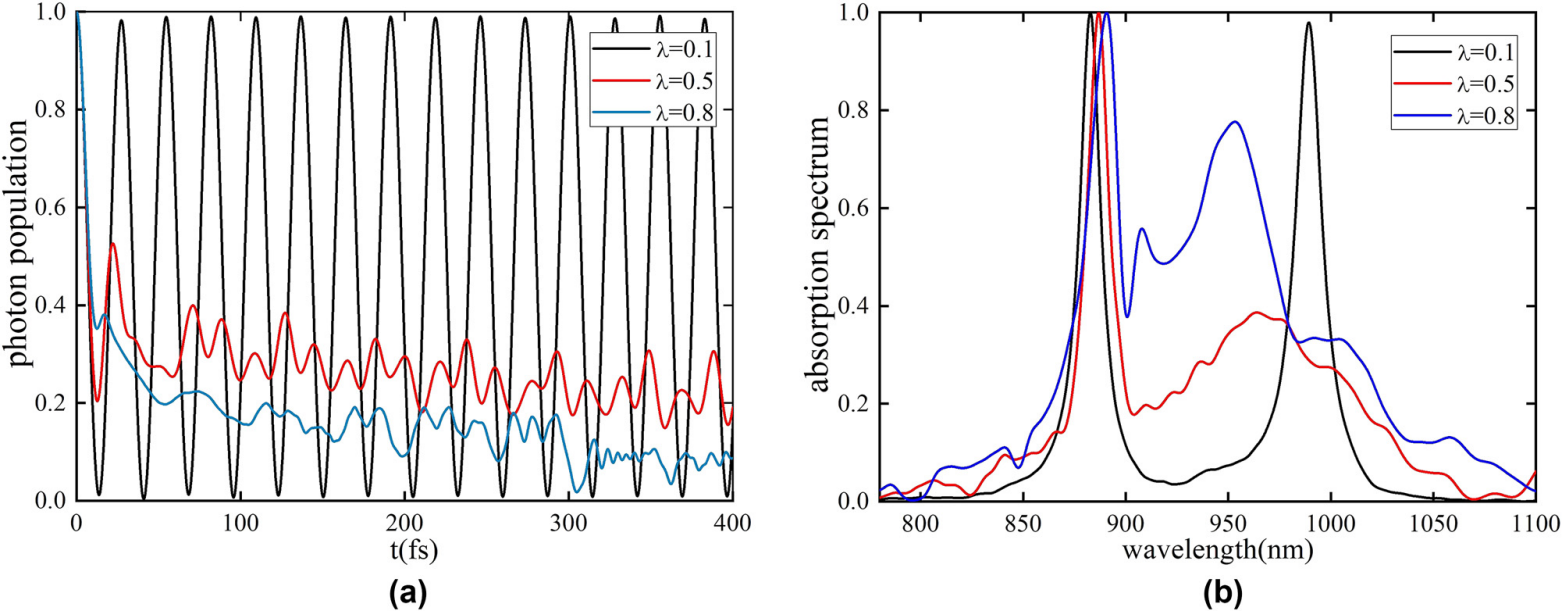
With the increase of phonon coupling strength, the dissipation effect of the system becomes significantly stronger. (a) The photon-mode population dynamics for *λ* = 0.1 (black), *λ* = 0.5 (red), and *λ* = 0.8 (blue). The stronger the phonon coupling strength, the more pronounced the dissipation effect in the photon distribution. (b) Absorption spectrum calculated for *λ* = 0.1 (black), *λ* = 0.5 (red), and *λ* = 0.8 (blue). The stronger the phonon coupling strength, the wider the linewidth of the LP peak in the absorption spectrum, which more clearly reflects the increase in system dissipation caused by phonon coupling.

Here we explore the strong cavity-system coupling regime (*g* > Γ). Below, a coupling coefficient of *g* = 0.15 eV and a center frequency of phonons *ω*_*k*0_ = 0.0496 eV are used. In the strong cavity-system coupling regime, where Rabi splitting occurs, phonon influence on the absorption spectrum is more pronounced than in the weak coupling regime. It can be seen from Figure 3(a) that when phonon coupling is taken into account, the photon population undergoes a dissipative dynamic process due to phonon coupling. At the same time, with the increase of phonon coupling, stronger dissipation is observed in the photon population. Additionally, in the Supplementary Materials Sections 3 and 4, we also discuss the calculation results and methods for the non-Hermitian Hamiltonian. As shown in Figure 3(b), as the phonon coupling strength increases, the shape of the UP peak remains unchanged, but the amplitude, position and width of the LP peak change significantly. The calculation satisfies the polaron decoupling threshold of the UP state (Ω ≫ *ω*_*k*0_) in this model [48]. As a result, the electronic and vibrational degrees of freedom of the UP state become separable, explaining the relative insensitivity of this absorption peak to varying phonon coupling [49], [50].

#### 3.2.2 Effect of the center phonon frequency

**Figure 4:**
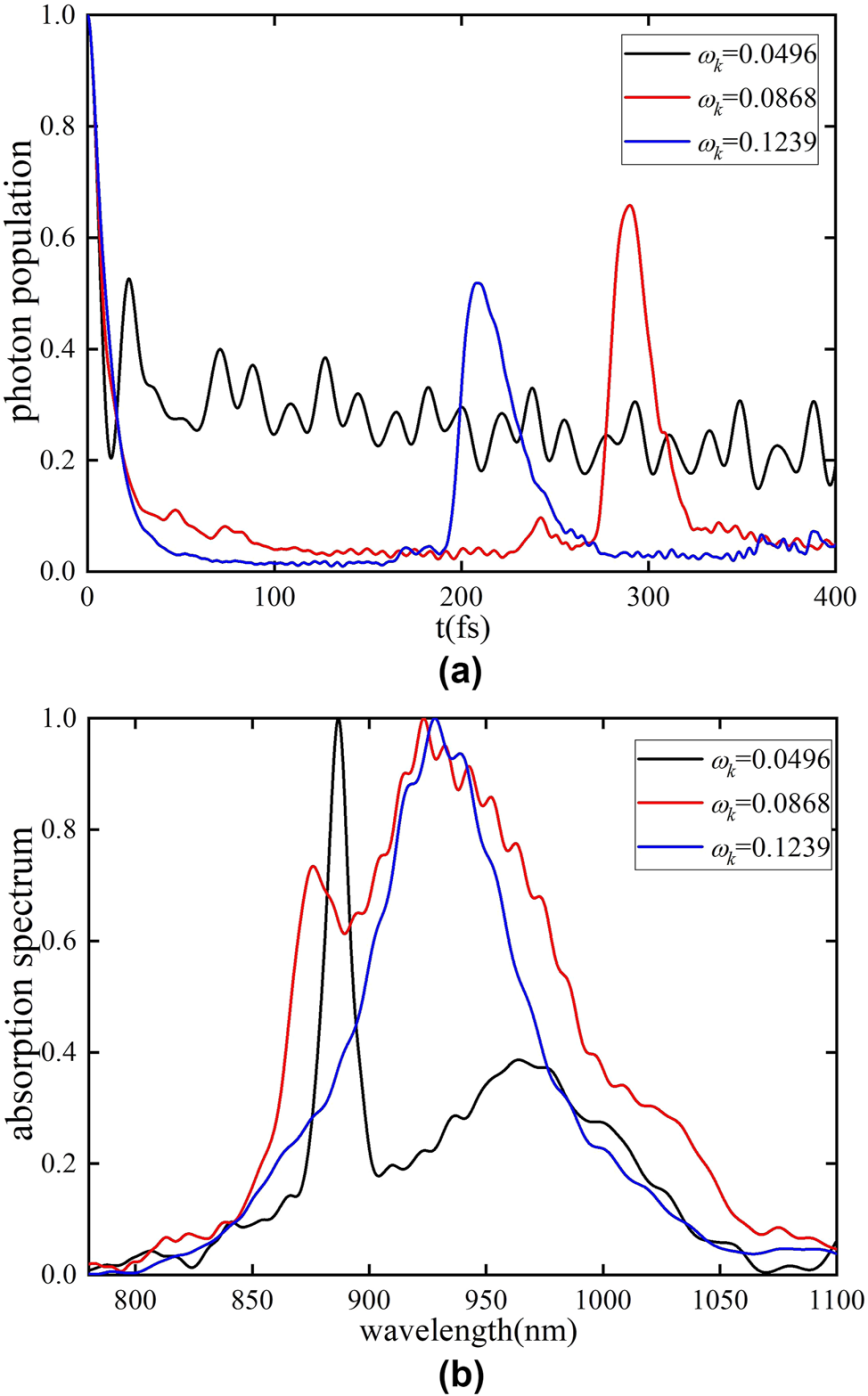
The dissipation effect induced by phonon coupling increases as the phonon center frequency increases. (a) The photon-mode population dynamics for *ω*_*k*0_ = 0.0496 eV (black), *ω*_*k*0_ = 0.0868 eV (red), and *ω*_*k*0_ = 0.1239 eV (blue). (b) Absorption spectrum calculated for *ω*_*k*0_ = 0.0496 eV (black), *ω*_*k*0_ = 0.0868 eV (red), and *ω*_*k*0_ = 0.1239 eV (blue).

Here we explore the role of the phonon sidebands via controlling the central frequency of the band. Below, a coupling coefficient of *g* = 0.15 eV, a center phonon frequency *ω*_*k*0_ = 0.0496 eV, 0.0868 eV and 0.1239 eV, and a phonon coupling strength *λ* = 0.5 are used. The three values of the center phonon energy *ω*_*k*0_ correspond to different metasurface materials such as metals and dielectrics. Figure 4(a) depicts the time evolution of the photon population. Since the phonon coupling will bring about a dissipative effect, it is obvious that the dissipation of the photon mode becomes larger as the center frequency of the phonon increases. The second-highest peaks in the red and blue curves of Figure 4(b) represent a recovery effect caused by the evolution of Rabi oscillations over time [51], [52], [53], and the dissipation of the system can also be seen according to the peak height. It is clear that under the same phonon coupling strength, the energy distribution of the phonon modes plays an even more important role in the dissipation process of the system. As shown in Figure 4(b), increasing the phonon energy range leads to a broader absorption linewidth at the same coupling strength. The blue absorption peak appears to be narrower, but the two peaks merge into a single peak. This means that the dissipation of the system is much greater than the system-cavity coupling (*g* < Γ), and the Rabi splitting vanishes, giving rise to a single peak, which highlights the phonons’ role in the dissipation process.

#### 3.2.3 Control of the coupling with the cavity mode

**Figure 5:**
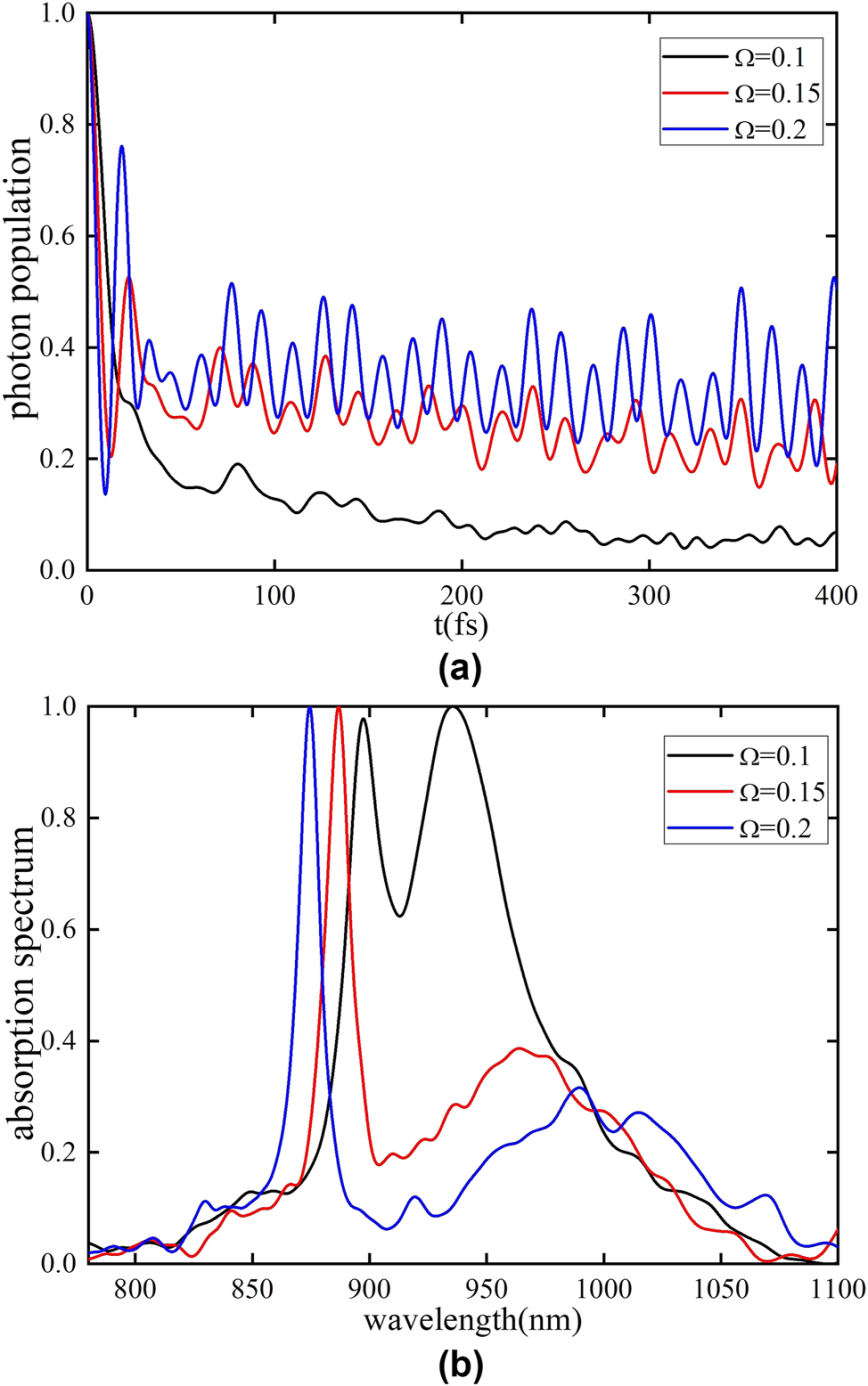
An increase in the Rabi frequency suppresses the dissipation rate of the system, but also enhances the energy exchange between cavity and system, leading to more phonon modes are effectively populated. (a) The photon-mode population dynamics for Ω = 0.1 (black), Ω = 0.15 (red), and Ω = 0.2 eV (blue). (b) Absorption spectrum calculated for Ω = 0.1 (black), Ω = 0.15 (red), and Ω = 0.2 eV (blue).

So far we have investigated the two limiting cases of weak and strong coupling to the cavity mode. We now explore the control of the system-cavity coupling and its effect on the transmission properties of the metasurface while fixing the parameters responsible for the phonon bath. Below, a center phonon frequency *ω*_*k*0_ = 0.0496 eV, and a phonon coupling strength *λ* = 0.5 are used. We investigate the influence of phonons on the spectrum under varying cavity Rabi splitting conditions. As previously discussed, phonon coupling brings about a dissipative effect, and in the case of constant phonon coupling strength, the stronger the coupling between the cavity and the system, the faster the light–matter energy exchange at a fixed phonon dissipation rate. Figure 5(a) shows that higher Rabi frequencies (indicating stronger cavity-system coupling) result in slower dissipation when phonon coupling remains constant. It is apparent that with the increase of coupling, the two Rabi splitting peaks become further separated, which is consistent with the Rabi splitting model (Figure 5(b)). Meanwhile, with the increase of Rabi splitting, the linewidth of the LP peak also widens. This is because the oscillation of photon population is a manifestation of coherent dynamics which can partly inhibit the dissipation process. If the Rabi frequency increases, the energy exchange between the system and the cavity accelerates, and at the same time, more phonon modes coupled to the system are effectively populated, which leads to the broadening of the absorption spectrum.

#### 3.2.4 Effect of the number of strong coupling phonon modes

**Figure 6:**
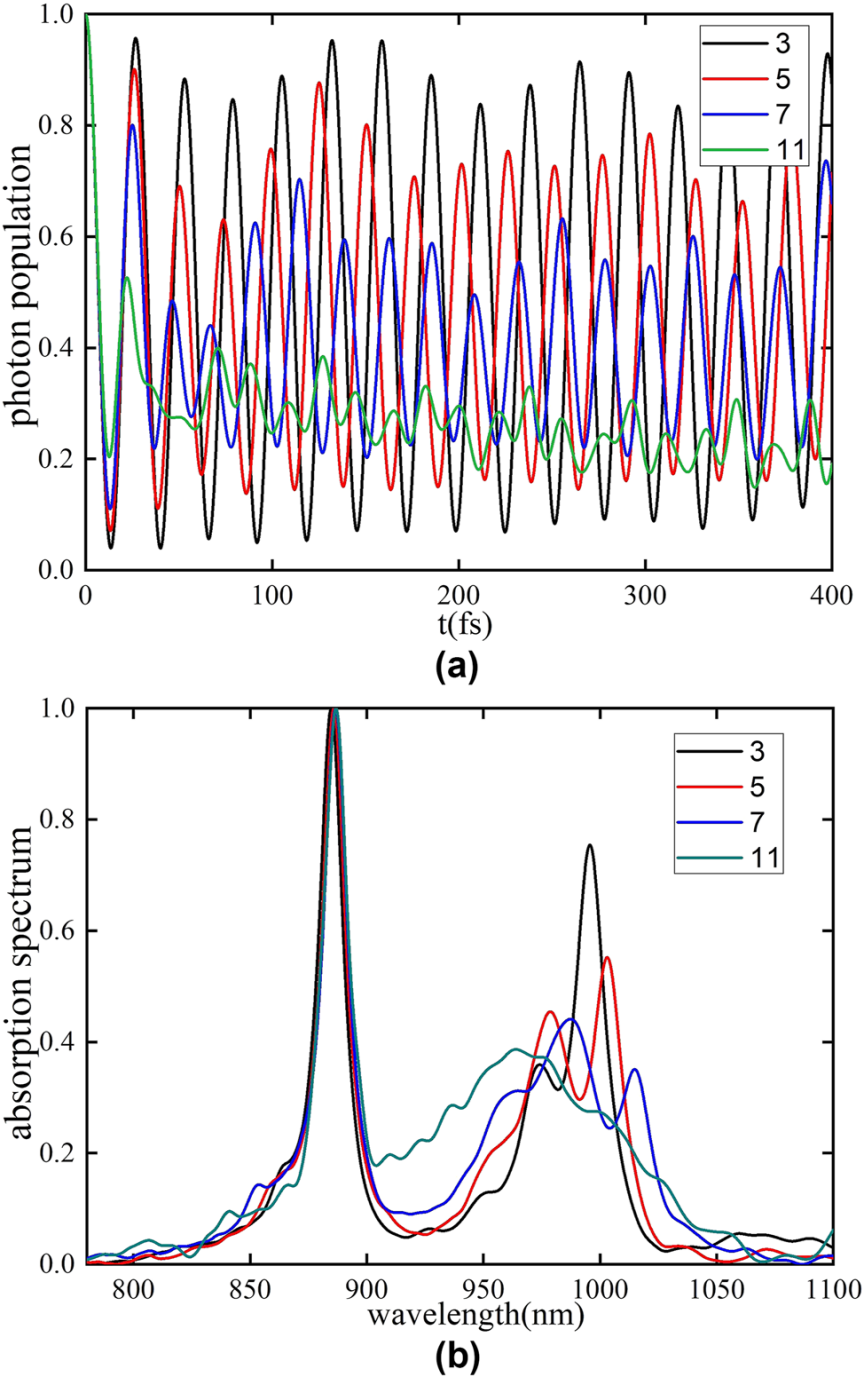
The larger the number of phonon modes with strong coupling near the center frequency, the more significant the impact on the system’s dissipation, which is also reflected in the widening linewidth of the absorption spectrum. (a) The evolution of photon modes with time for different strongly coupled phonon numbers under strong cavity-system coupling. (b) The absorption peaks of different strongly coupled phonon numbers.

Thus far, we have assumed that the system is coupled to all the phonon modes with an equal coupling strength. In contrast, in real systems, microscopic theory suggests that a realistic bath spectral density determines the number of phonon modes that strongly couple with the system, while the remaining modes exhibit weak coupling. Below we explore how this number of strong coupling modes affects the metasurface transmission properties and examine the impact of their energy distribution relative to the phonon band center. Here, a coupling coefficient of *g* = 0.15 eV and a center phonon frequency *ω*_*k*0_ = 0.0496 eV are used. Varying numbers of strong-coupling phonon modes (*λ* = 0.5) in the vicinity of *ω*_*k*0_ are selected (such as 3, 5, and 7 modes), while the remaining phonon modes are weak coupled to the system (*λ* = 0.1). As the number of strong coupling phonon modes increases, the dissipative behavior of the system becomes more pronounced (Figure 6(a)). With fewer strong coupling phonon modes, the system’s linewidth narrows significantly, indicating a reduction in dissipation (Figure 6(b)). As the number of strong coupling phonon modes increases, the linewidth of the absorption peak broadens due to energy redistribution into vibrational levels associated with strong coupling phonons.

**Figure 7:**
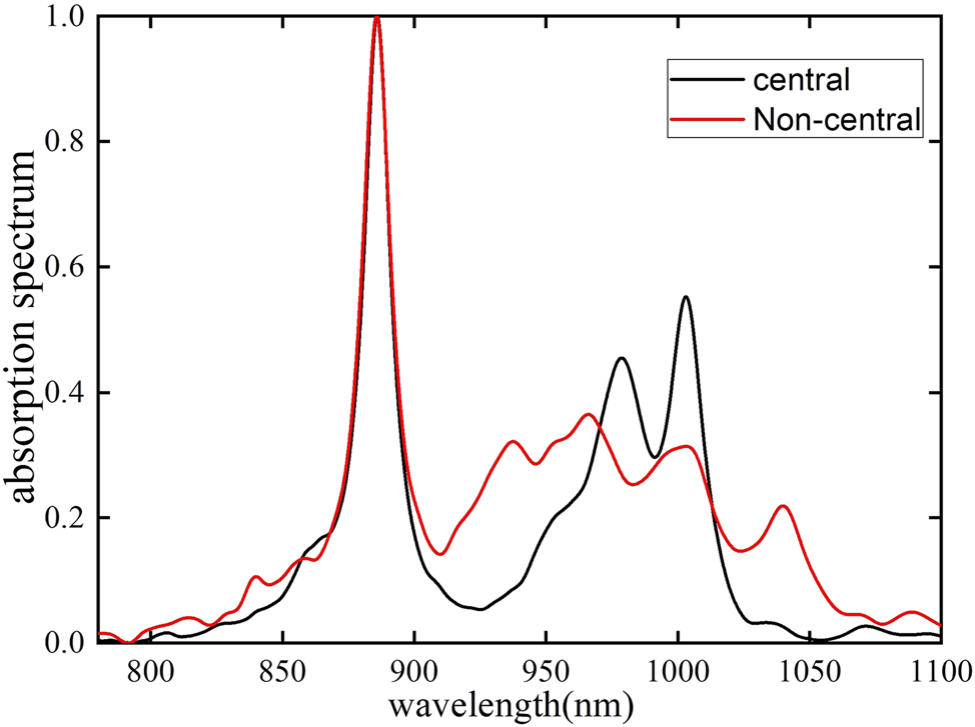
The absorption spectrum of the same number of strong coupling phonons in the central and non-central regions. For the same number of phonon modes, those with strong coupling near the phonon center frequency cause less dissipation compared to those with strong coupling at off-center frequencies, leading to a narrower linewidth in the absorption spectrum.

As already seen in the aforementioned discussion, if the phonon modes are strongly coupled at the center frequency *ω*_*k*0_, the linewidth of the absorption spectrum is greatly reduced as compared to the case of strong coupling of every phonon modes. In order to explore the effect of the strong coupling of non-center frequency phonon modes (i.e., far from *ω*_*k*0_) on the absorption spectrum, we chose the number of strong coupling phonon modes as 5, which makes the proportion of strong coupling and weak coupling modes approximately the same. It is found that when the selected phonon modes are strongly coupled at the non-center frequency, the absorption spectrum broadens compared to coupling at the center frequency (Figure 7). This can be attributed to the vibrational levels generated by phonon modes strongly coupled in regions distant from the phonon bath center frequency, facilitating greater energy exchange between the photon and vibrational energy levels in those regions, which ultimately broadens the absorption spectrum linewidth.

## 4 Conclusions

In this study, we have examined the influence of the phonon environment on the transmission properties of metasurfaces. We explore both weak and strong coupling to the phonon bath, as well as the interaction between the metasurface cavity and the material system. Using MD2A, we conducted a thorough analysis of the dynamics of the JC model with a phonon bath, investigating the combined effects of varying phonon coupling strengths and different numbers of strongly coupled phonon modes on the photonic mode population and absorption spectrum *F*(*ω*) in both weak and strong light–matter coupling regimes. Our findings reveal that phonons primarily influence the dissipative behavior of the system. This effect is significantly more pronounced under strong phonon coupling than under weak coupling and has a substantial impact on the linewidth of the absorption spectrum.

It is found that in the weak light–matter coupling regime, phonon coupling has a minimal impact on the linewidth of the absorption spectrum, indicating that phonons have little effect on the system’s dissipation under weak coupling. In contrast, in the strong light–matter coupling regime, the strength of phonon coupling directly influences the system’s dissipation, with stronger phonon coupling leading to a more significant dissipation effect. Meanwhile, the central frequency of the phonon bath also affects the system dissipation. The higher the central frequency of the phonon bath, the greater the dissipation of the system. Furthermore, a higher number of strong-coupling phonon modes in the vicinity of the center phonon frequency leads to stronger overall dissipation of the system, resulting in a larger linewidth of the absorption spectrum. If the total dissipation (including both phonon-induced dissipation and intrinsic system dissipation) is much greater than the Rabi frequency, the absorption may merge into one peak, which erodes the Rabi splitting. Therefore, in the strong light–matter coupling regime, phonon coupling exerts a substantial influence on system dissipation.

In conclusion, we have developed numerically accurate methods for simulating the transmission properties of the metasurfaces based on dynamics of the JC model with a phonon bath, providing qualitative insights into how different phonon states influence system dissipation. This advances the understanding and modeling of the dynamics and spectral responses of polarized metasurfaces. Furthermore, we demonstrate that the absorption spectrum under strong light–matter coupling is highly sensitive to phonon states, emphasizing the importance of selecting appropriate materials for phonon coupling in detecting strong coupling effects. With the rise of artificial intelligence, metasurfaces are increasingly being designed with intelligent approaches. When incorporating temperature as an additional variable in our model, it becomes theoretically possible to verify experimental observations of Rabi splitting in metasurfaces at room temperature [27], [28], [29], [30]. Although the phonon characteristics of metasurfaces have been explored in the context of nonlinear processes, as noted in previous studies [32], this work focuses on phonon coupling effects in linear systems. This is a continous effort to go deeper in microscopic properties of metasurfaces starting with mesoscopic theory developed for linear response calculations [54], nonlinear response [55], quantum state engineering [56], full polarization control in staggered geometry [57]. This extends the potential applications of metasurfaces and enhances our understanding of their quantum properties.

## Supplementary Material

Supplementary Material Details
