# Supplementary material for: Phonon controlled transmission properties of metasurfaces under strong light–matter coupling
Source: Nanophotonics. 2025 May 26;14(13):2345–54. doi: 10.1515/nanoph-2025-0123 (PMC12199562; doi:10.1515/nanoph-2025-0123)
Supplement: Supplementary file 1 — Supplementary Material Details [file j_nanoph-2025-0123_suppl_001.pdf]

Research Article

Zengshun Jiang, Kewei Sun, Yang Zhao*, and Konstantin Dorfman*

Phonon controlled transmission properties of metasurfaces under strong light-matter coupling

1 S1 FDTD Simulations

The full-wave simulations of SLR metasurface absorption spectrum signal were performed with the Lumerical FDTD Software. The size of Au nanoparticles was diameter D=65 nm and height H=60 nm, surrounded by a high refractive index medium (n=1.52), and the period was chosen to be 550 nm along both the x- and y-axis directions (Figure S1). The periodic boundary conditions were used along x- and y- directions to simulate the array of meta-atoms. The perfect-matched layer conditions were applied in z-directions. And the electric field at the resonance wavelength in the xy-plane is shown in Figure S2. It is find that the electric field is symmetrically distributed on both sides of the cylinder, the same as in a dipolar manner. The permittivity of gold used in the simulations was taken from [1].

Zengshun Jiang, State Key Laboratory of Precision Spectroscopy, East China Normal University, Shanghai 200241, China, 15863450519@163.com
Kewei Sun, School of Science, Hangzhou Dianzi University, Hangzhou 310018, China, skw79724@hdu.edu.cn; https://orcid.org/0000-0003-1542-8352
***Corresponding author: Yang Zhao,** School of Materials Science and Engineering, Nanyang Technological University, Singapore 639798, Singapore, yzhao@ntu.edu.sg; https://orcid.org/0000-0002-7916-8687
***Corresponding author: Konstantin Dorfman,** State Key Laboratory of Precision Spectroscopy, East China Normal University, Shanghai 200241, China; Center for Theoretical Physics and School of Physics and Optoelectronic Engineering, Hainan University, Haikou 570228, China; and Himalayan Institute for Advanced Study, Unit of Gopinath Seva Foundation, MIG 38, Avas Vikas, Rishikesh 249201, Uttarakhand, India, dorfmank@hainanu.edu.cn; https://orcid.org/0000-0001-9963-0878

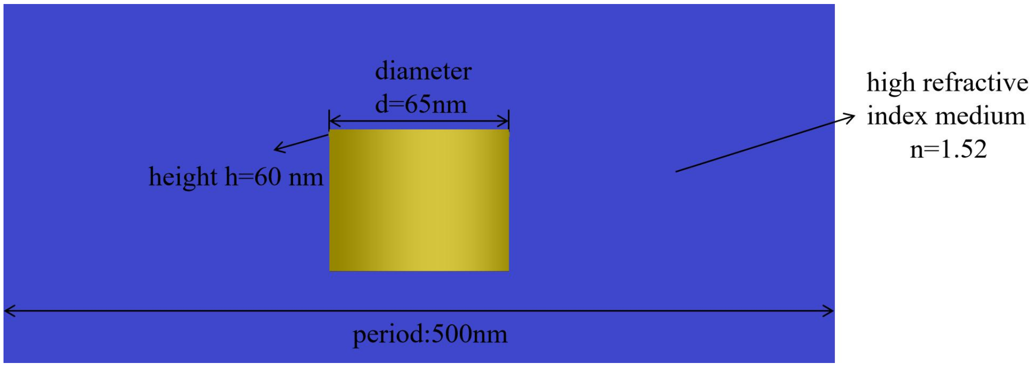

Fig. 1: Schematic diagram of the unit cell

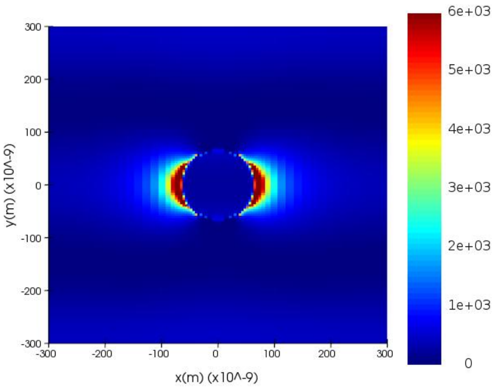

Fig. 2: The calculated 2D electric-field intensity distribution for the bare plasmonic metasurface in top–down view xy-plane. The diameter of the single nano-pillar is 65 nm.

2 Equations of Motion Based on Multi-D2 Ansatz (Hermitian Hamiltonian)

The time-dependent variational parameters, i.e., $A_{mn}(t)$, and $f_{mk}(t)$ are determined via the variational principle[2–4]

$$\frac{d}{dt}\frac{\partial L}{\partial \dot{\mu}_i^*} - \frac{\partial L}{\partial \mu_i^*} = 0 \tag{1}$$

where the Lagrangian L is given by

$$L = \frac{i}{2}\left[\left\langle D_2^M(t)\middle|\frac{\overrightarrow{\partial}}{\partial t}\middle|D_2^M(t)\right\rangle - \left\langle D_2^M(t)\middle|\frac{\overleftarrow{\partial}}{\partial t}\middle|D_2^M(t)\right\rangle\right] - \left\langle D_2^M(t)\middle|H\middle|D_2^M(t)\right\rangle =: L_{td} - L_H. \tag{2}$$

Using the normalization of the Davydov Ansatz, the derivative needs only to be taken to the right, simplifying the derivation considerably. Thus the first term in Eq. (S2), L_{td}, is given by

$$\begin{aligned} L_{td} &= i\left\langle D_2^M(t)\middle|\frac{\overrightarrow{\partial}}{\partial t}\middle|D_2^M(t)\right\rangle \\ &= i\sum_n\sum_{m,m'=1}^M A_{mn}^* S_{mm'}\left(\dot{A}_{m'n} + A_{m'n}\sum_k \frac{2f_{mk}^*\dot{f}_{m'k} - f_{m'k}^*\dot{f}_{mk} - f_{m'k}\dot{f}_{mk}^*}{2}\right). \end{aligned} \tag{3}$$

The second term L_H is in fact the average energy of the system in Eq. (S2), which reads

$$L_H = \left\langle D_2^M(t)\middle| H \middle| D_2^M(t)\right\rangle \tag{4}$$

It follows that the time-dependent variational principle results in equations of motion for $A_{m'0}$

$$\begin{aligned} & i\sum_{m'=1}^M \dot{A}_{m'0}S_{mm'} + i\sum_{m'=1}^M A_{m'0}\sum_k\left[-\frac{1}{2}\left(\dot{f}_{m'k}f_{m'k}^* + f_{m'k}\dot{f}_{m'k}^*\right) + f_{mk}^*\dot{f}_{m'k}\right]S_{mm'} \\ &= \omega_c\sum_{m'=1}^M \dot{A}_{m'0}S_{mm'} + \sum_{m'=1}^M\sum_{n=1}^N gA_{m'n}S_{mm'} + \sum_{m'=1}^M A_{m'0}\sum_k \omega_k f_{mk}^* f_{m'k}S_{mm'} \end{aligned} \tag{5}$$

and for $A_{m'n}$,

$$\begin{aligned} & i\sum_{m'=1}^M \dot{A}_{m'n}S_{mm'} + i\sum_{m'=1}^M A_{m'n}\sum_k\left[-\frac{1}{2}\left(\dot{f}_{m'k}f_{m'k}^* + f_{m'k}\dot{f}_{m'k}^*\right) + f_{mk}^*\dot{f}_{m'k}\right]S_{mm'} \\ &= \omega_n\sum_{m'=1}^M \dot{A}_{m'n}S_{mm'} + \sum_{m'=1}^M\sum_{n=1}^N gA_{m'0}S_{mm'} + \sum_{m'=1}^M A_{m'n}\sum_k \omega_k f_{mk}^* f_{m'k}S_{mm'} \\ &- \lambda\sum_{m'=1}^M\sum_k \omega_k A_{m'n}(f_{m'k} + f_{mk}^*)S_{mm'} \end{aligned} \tag{6}$$

Similarly, the equations of motion for f_{mk} are

$$
\begin{aligned}
& i\sum_{n=1}^{N}\sum_{m'=1}^{M} A_{mn}^{*}\dot{A}_{m'n}f_{m'k}S_{mm'} + i\sum_{n=1}^{N}\sum_{m'=1}^{M} A_{mn}^{*}A_{m'n}f_{m'k}S_{mm'} \\
& + i\sum_{n=1}^{N}\sum_{m'=1}^{M} A_{mn}^{*}A_{m'n}f_{m'k}S_{mm'}\sum_{k}\left[f_{mk'}^{*}\dot{f}_{m'k'} - \frac{1}{2}\left(\dot{f}_{m'k'}f_{m'k'}^{*} + f_{m'k'}\dot{f}_{m'k'}^{*}\right)\right] \\
& = \omega_c\sum_{m'=1}^{M} A_{m0}^{*}A_{m'0}f_{m'k}S_{mm'} + \sum_{n=1}^{N}\sum_{m'=1}^{M}\omega_n A_{mn}^{*}A_{m'n}f_{m'k}S_{mm'} \\
& + \sum_{m'=1}^{M}\sum_{n=1}^{N} g(A_{mn}^{*}A_{m'0}+A_{m0}^{*}A_{m'n})f_{m'k}S_{mm'} + \sum_{n=1}^{N}\sum_{m'=1}^{M} A_{mn}^{*}A_{m'n}\omega_k f_{m'k}S_{mm'} \\
& + \sum_{n=1}^{N}\sum_{m'=1}^{M} f_{m'k}\sum_{k} A_{mn}^{*}A_{m'n}\omega_k f_{mk'}^{*} f_{m'k'} S_{mm'} - \lambda\sum_{n=1}^{N}\sum_{m'=1}^{M}\sum_{k}\omega_k A_{mn}^{*}A_{m'n}S_{mm'} \\
& - \lambda\sum_{n=1}^{N}\sum_{m'=1}^{M} f_{m'k}\sum_{k'}\omega_{k'}A_{mn}^{*}A_{m'n}(f_{m'k'} + f_{mk'}^{*})S_{mm'} \qquad (7)
\end{aligned}
$$

3 Equations of Motion Based on Multi-D2 Ansatz (non-Hermitian Hamiltonian)

Calculating the Hermitian Hamiltonian requires an additional broadening (in Eq. (11)) to replace the dissipation of the system when calculating the absorption spectrum. However the non-Hermitian Hamiltonian does not require this additional broadening. Here we introduce a non-Hermitian Hamiltonian, which can be expressed as

$$
\hat{H} = \hbar(\omega_0 - i\Gamma)\hat{\sigma}^{\dagger}\hat{\sigma}^{-} + \hbar\omega_C\hat{a}^{\dagger}\hat{a} + \hbar g(\hat{a}^{\dagger}\hat{\sigma}^{-} + \hat{a}\hat{\sigma}^{\dagger}) + \sum_{k}\hbar\omega_k\hat{b}_k^{\dagger}\hat{b}_k + \sum_{k}\hbar\omega_k\lambda\hat{\sigma}^{\dagger}\hat{\sigma}^{-}(\hat{b}_k^{\dagger} + \hat{b}_k) \qquad (8)
$$

It follows that the time-dependent variational principle results in equations of motion for $A_{m'0}$

$$
\begin{aligned}
& i\sum_{m'=1}^{M}\dot{A}_{m'0}S_{mm'} + i\sum_{m'=1}^{M} A_{m'0}\sum_{k}\left[-\frac{1}{2}\left(\dot{f}_{m'k}f_{m'k}^{*} + f_{m'k}\dot{f}_{m'k}^{*}\right) + f_{mk}^{*}\dot{f}_{m'k}\right]S_{mm'} \\
& = \omega_c\sum_{m'=1}^{M}\dot{A}_{m'0}S_{mm'} + \sum_{m'=1}^{M}\sum_{n=1}^{N} gA_{m'n}S_{mm'} + \sum_{m'=1}^{M} A_{m'0}\sum_{k}\omega_k f_{mk}^{*}f_{m'k}S_{mm'} \qquad (9)
\end{aligned}
$$

and for $A_{m'n}$,

$$i\sum_{m'=1}^{M}\dot{A}_{m'n}S_{mm'}+i\sum_{m'=1}^{M}A_{m'n}\sum_{k}\left[-\frac{1}{2}\left(\dot{f}_{m'k}f^{*}_{m'k}+f_{m'k}\dot{f}^{*}_{m'k}\right)+f^{*}_{mk}\dot{f}_{m'k}\right]S_{mm'}$$
$$=(\omega_n-i\Gamma)\sum_{m'=1}^{M}\dot{A}_{m'n}S_{mm'}+\sum_{m'=1}^{M}\sum_{n=1}^{N}gA_{m'0}S_{mm'}+\sum_{m'=1}^{M}A_{m'n}\sum_{k}\omega_k f^{*}_{mk}f_{m'k}S_{mm'}$$
$$-\lambda\sum_{m'=1}^{M}\sum_{k}\omega_k A_{m'n}(f_{m'k}+f^{*}_{mk})S_{mm'} \quad (10)$$

Similarly, the equations of motion for f_{mk} are

$$i\sum_{n=1}^{N}\sum_{m'=1}^{M}A^{*}_{mn}\dot{A}_{m'n}f_{m'k}S_{mm'}+i\sum_{n=1}^{N}\sum_{m'=1}^{M}A^{*}_{mn}A_{m'n}f_{m'k}S_{mm'}$$
$$+i\sum_{n=1}^{N}\sum_{m'=1}^{M}A^{*}_{mn}A_{m'n}f_{m'k}S_{mm'}\sum_{k}\left[f^{*}_{mk'}\dot{f}_{m'k'}-\frac{1}{2}\left(\dot{f}_{m'k'}f^{*}_{m'k'}+f_{m'k'}\dot{f}^{*}_{m'k'}\right)\right]$$
$$=\omega_c\sum_{m'=1}^{M}A^{*}_{m0}A_{m'0}f_{m'k}S_{mm'}+\sum_{n=1}^{N}\sum_{m'=1}^{M}(\omega_n-i\Gamma)A^{*}_{mn}A_{m'n}f_{m'k}S_{mm'}$$
$$+\sum_{m'=1}^{M}\sum_{n=1}^{N}g(A^{*}_{mn}A_{m'0}+A^{*}_{m0}A_{m'n})f_{m'k}S_{mm'}+\sum_{n=1}^{N}\sum_{m'=1}^{M}A^{*}_{mn}A_{m'n}\omega_k f_{m'k}S_{mm'}$$
$$+\sum_{n=1}^{N}\sum_{m'=1}^{M}f_{m'k}\sum_{k}A^{*}_{mn}A_{m'n}\omega_k f^{*}_{mk'}f_{m'k'}S_{mm'}-\lambda\sum_{n=1}^{N}\sum_{m'=1}^{M}\sum_{k}\omega_k A^{*}_{mn}A_{m'n}S_{mm'}$$
$$-\lambda\sum_{n=1}^{N}\sum_{m'=1}^{M}f_{m'k}\sum_{k'}\omega_{k'}A^{*}_{mn}A_{m'n}(f_{m'k'}+f^{*}_{mk'})S_{mm'} \quad (11)$$

The absorption spectrum is now given by:

$$F(\omega)=\frac{1}{\pi}\Re\int_{0}^{\infty}F(t)e^{-(\gamma'-i\omega)t}dt$$
$$=\frac{1}{\pi}\Re\int_{0}^{\infty}dt\sum_{m,m'=1}^{M}A^{*}_{m'1c}(0)A_{m1c}(t)e^{-(\gamma'-i\omega)t}$$
$$\times e^{\sum_k\left(f^{*}_{m'k}(0)f_{m'k}(t)+\dot{f}^{*}_{m'k}(0)\dot{f}_{m'k}(t)\right)} \quad (12)$$

4 Camparison between non-Hermitian Hamiltonian and Hermitian Hamiltonian model in Multi-D2 Ansatz

Fig. 3a shows the time evolution of the photon population calculated using the current model, with the same parameters as in Fig. 3a of the main text. By comparing it with Fig.

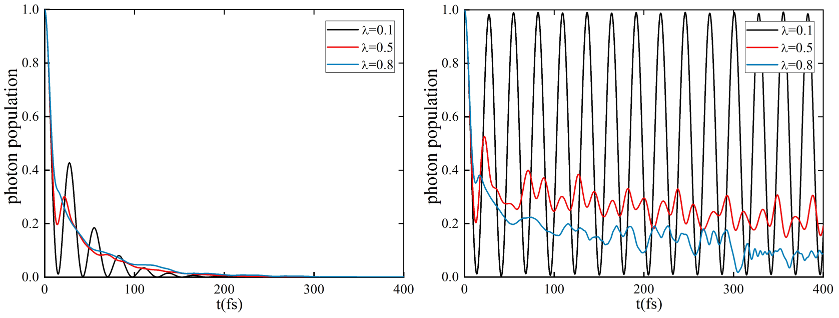

(a) **(b)**

Fig. 3: (a) The photon-mode population dynamics for $\lambda = 0.1$ (black), $\lambda = 0.5$ (red), and $\lambda = 0.8$(blue) in non-Hermitian Hamiltonian model. (b)Calculation results based on the Hermitian Hamiltonian without dissipation (Fig. 3a in the main text)

3b, it is observed that under the non-Hermitian Hamiltonian model (which accounts for dissipation), the effect of phonon coupling is not easily detectable. However, even when different models are used to calculate the absorption spectrum, the results remain identical. Therefore, the main text adopts a Hermitian Hamiltonian for the calculations and introduces a phenomenological dissipation factor only in the final step when computing the absorption spectrum.

References

[1] Peter B Johnson and R-WJPrB Christy. Optical constants of the noble metals. *Physical review B*, 6(12):4370, 1972.

[2] Ke-Wei Sun, Maxim F Gelin, Vladimir Y Chernyak, and Yang Zhao. Davydov ansatz as an efficient tool for the simulation of nonlinear optical response of molecular aggregates. *The Journal of Chemical Physics*, 142(21), 2015.

[3] Kewei Sun, Weiwei Xie, Lipeng Chen, Wolfgang Domcke, and Maxim F Gelin. Multi-faceted spectroscopic mapping of ultrafast nonadiabatic dynamics near conical intersections: A computational study. *The Journal of Chemical Physics*, 153(17), 2020.

[4] Yang Zhao, Kewei Sun, Lipeng Chen, and Maxim Gelin. The hierarchy of davydov's ansätze and its applications. *Wiley Interdisciplinary Reviews: Computational Molecular Science*, 12(4):e1589, 2022.
